# Sialic acid-dependent cell entry of human enterovirus D68

**DOI:** 10.1038/ncomms9865

**Published:** 2015-11-13

**Authors:** Yue Liu, Ju Sheng, Jim Baggen, Geng Meng, Chuan Xiao, Hendrik J. Thibaut, Frank J. M. van Kuppeveld, Michael G. Rossmann

**Affiliations:** 1 Department of Biological Sciences, Hockmeyer Hall of Structural Biology, 240 South Martin Jischke Drive, Purdue University, West Lafayette, Indiana 47907, USA; 2 Department of Infectious Diseases and Immunology, Virology Division, Faculty of Veterinary Medicine, Utrecht University, 3584CL Utrecht, The Netherlands; 3 Department of Chemistry, University of Texas at El Paso, 500 W. University Avenue, El Paso, Texas 79968-0519, USA

## Abstract

Human enterovirus D68 (EV-D68) is a causative agent of childhood respiratory diseases and has now emerged as a global public health threat. Nevertheless, knowledge of the tissue tropism and pathogenesis of EV-D68 has been hindered by a lack of studies on the receptor-mediated EV-D68 entry into host cells. Here we demonstrate that cell surface sialic acid is essential for EV-D68 to bind to and infect susceptible cells. Crystal structures of EV-D68 in complex with sialylated glycan receptor analogues show that they bind into the ‘canyon' on the virus surface. The sialic acid receptor induces a cascade of conformational changes in the virus to eject a fatty-acid-like molecule that regulates the stability of the virus. Thus, virus binding to a sialic acid receptor and to immunoglobulin-like receptors used by most other enteroviruses share a conserved mechanism for priming viral uncoating and facilitating cell entry.

## Abstract (web summary)

The human enterovirus D68 (EV-D68) is a causative agent of childhood respiratory infections, but despite its prevalence the exact mechanism mediating its cell entry have not been fully established. Here, the authors show how EV-D68 binds to sialic acid on the cell surface to initiate infection.

## Graphical abstract

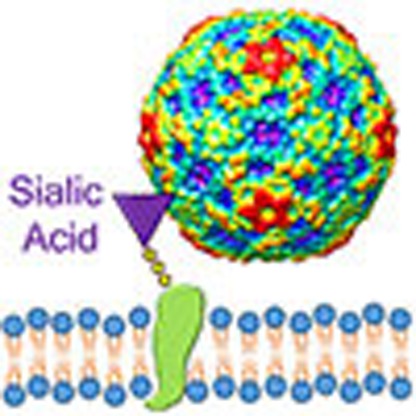

Piconaviruses are a family of non-enveloped, icosahedral, positive-stranded RNA viruses. Enteroviruses (EV), an important genus of this family, include a variety of human pathogens^1^, such as polioviruses and the human enterovirus D68 (EV-D68). EV-D68 is a causative agent of childhood respiratory infections^2^ and occasionally leads to neurological diseases^3^. Recent outbreaks of EV-D68, including the 2014 outbreak in the United States^4^, indicate that this virus has now emerged as a global public health threat^5^. However, the tissue tropism and pathogenesis of EV-D68 is poorly understood. Specifically, there is a lack of knowledge on the receptor-mediated EV-D68 entry into host cells.

The capsid of EVs consist of 60 copies of each of the viral proteins VP1, VP2 and VP3, that form an icosahedral shell with pseudo T=3 symmetry, and 60 copies of VP4 that form a network on the interior surface of the capsid^6^,^7^. A depression on the virion surface, the canyon, that encircles each five-fold axis, has limited accessibility to neutralizing antibodies^6^. This region is frequently the binding site for immunoglobulin-like (Ig-like) receptors^8^. In most EV structures, a hydrophobic pocket in VP1 is located underneath the canyon and accommodates a ‘pocket factor', which is a fatty-acid like molecule that stabilizes the virion^9^,^10^,^11^. Many EVs interact with their cognate Ig-like receptors in a two-step mechanism^12^,^13^, which involves a competition between the pocket factor and the receptor^10^,^14^. The binding of an Ig-like receptor at physiological temperatures leads to an irreversible conformational switch to an expanded A (altered) particle^15^,^16^, an intermediate during viral uncoating^17^. Binding of Ig-like receptors facilitates virus entry and subsequently genome release into the cell cytosol for successful viral replication. Thus, a series of capsid-binding inhibitors (for example, pleconaril^18^), which replace the pocket factor in binding EVs^19^,^20^, were developed to inhibit EV infections by interfering with cell entry of EVs. Carbohydrate receptors, including sialic acid and heparin sulfate, have also been identified as receptors for a number of EVs^21^,^22^,^23^,^24^,^25^,^26^. Nevertheless, it is not known whether these can also initiate virus uncoating to facilitate virus infection.

EV-D68 belongs to the poorly characterized species EV-D. However, it had been reported that EV-D70, a close relative of EV-D68, uses sialic acid as a cellular receptor^21^,^27^. Furthermore, a glycan array analysis showed that EV-D68 can bind to synthetic glycoproteins with a terminally linked sialic acid^28^. In addition, sialic acid terminated molecules are widely distributed and abundantly expressed in the human respiratory tract^29^.

Here we identify sialic acid as a receptor of EV-D68 and show that sialylated glycans bind to the virus ‘canyon'. We also show that binding of sialic acid causes a series of conformational changes that destabilize the virus by ejection of the ‘pocket factor' to initiate infection.

## Results

### Identification of sialic acid as a receptor for EV-D68

**Figure 1:**
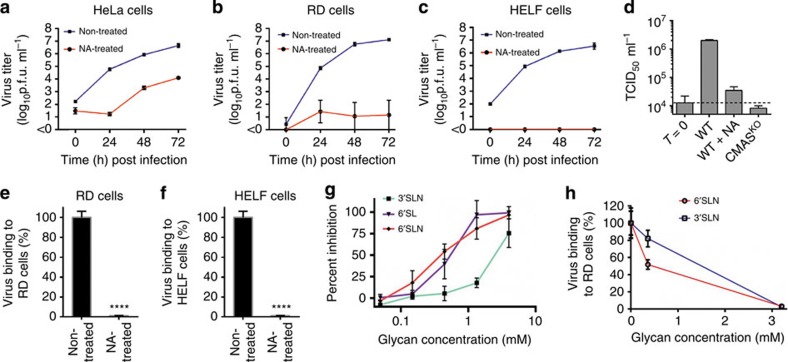
Cell surface sialic acid has a crucial role in EV-D68 (strain Fermon CA62-1) attachment and infection. (**a**–**c**) Growth curve of EV-D68 in susceptible cell lines cells. Neuraminidase treatment of susceptible cell lines inhibits EV-D68 infection. (**d**) HAP1 cells (WT and CMAS-KO) were infected with EV-D68 at an MOI of 1 and virus titres were determined at 0 h (T=0) or 10-h post infection. (**e**,**f**) Histograms showing virus binding to susceptible cells. *P*<0.0001 by Student's *t*-test. (**g**) Preincubation of 6′SL, 6′SLN or 3′SLN with EV-D68 prevents killing of RD cells caused by virus infection. (**h**) Preincubation of 6′SLN or 3′SLN with EV-D68 inhibits viral attachment to RD cells. All data are represented as mean±s.d. Experiments were performed at least in triplicate.

The possible use of sialic acid as a cellular receptor by EV-D68 was examined by performing attachment and infectivity assays. We found that removal of cell surface sialic acid by neuraminidase treatment of HeLa cells, human rhabdomyosarcoma (RD) cells and human lung embryonic fibroblast (HELF) cells significantly reduced infectivity of the EV-D68 Fermon prototype strain (Fig. 1a–c). These results suggest that sialic acid might be a functional receptor in these cell lines. To obtain further evidence that sialic acid is a functional receptor for EV-D68, human HAP1 cells were used in which the sialic acid activating enzyme cytidine monophosphate *N*-acetylneuraminic acid synthase (CMAS) had been knocked out. These cells are devoid of sialic acids on their surface and had previously been shown to be highly resistant to influenza A virus infection^30^. We found that these cells were resistant to EV-D68 (Fermon strain) infection, further confirming the importance of sialic acid for EV-D68 infection (Fig. 1d). Furthermore, neuraminidase treatment of RD and HELF cells led to significantly decreased virus attachment (Fig. 1e,f).

In view of these results as well as the previous glycan array analysis^29^, and also because *N*-acetylneuraminic acid (Neu5Ac) is a frequently occurring sialic acid in humans, the sialylated trisaccharides Neu5Acα2-3Galβ1-4GlcNAc (3′SLN), Neu5Acα2-6Galβ1-4GlcNAc (6′SLN), and Neu5Acα2-6Galβ1-4Glc (6′SL) were used as potential receptor analogues. Preincubation of EV-D68 with these sialylated trisaccharides inhibited viral attachment and prevented killing of RD cells in a concentration-dependent manner (Fig. 1g,h). Therefore, sialic acid has a crucial role in EV-D68 attachment and infection.

### The binding site of sialylated receptor analogues

**Figure 2:**
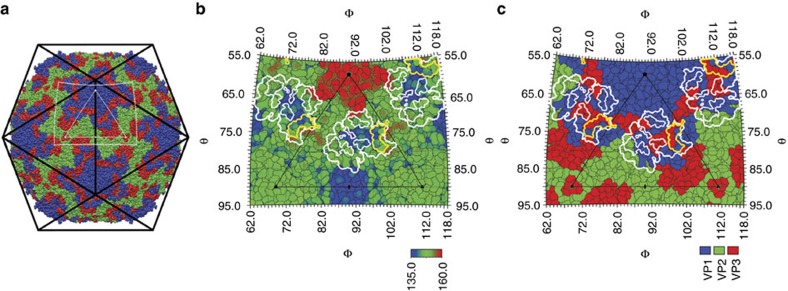
Sialylated receptor analogues bind to the EV-D68 canyon. (**a**) An EV-D68 virus particle in an icosahedral cage (black). The white triangle represents one icosahedral asymmetric unit. The surrounding white rectangular outline represents the limits of the figures shown in (**b**,**c**). The thick white contour outlines the summation of five superimposed footprints of Ig-like receptors on the virus, whereas the thinner white contour represents the consensus footprint of at least four footprints. Shown also is the footprint of the sialylated trisaccharides (yellow). The background is a map of the EV-D68 surface residues coloured by polypeptide in (**a**,**c**) or coloured by radial distance in Å from the virus centre in (**b**).

The crystal structures were determined of EV-D68 when in complex with 3′SLN, 6′SL or 6′SLN. The resolution of these structures ranged from 2.2 to 2.3 Å (Supplementary Table 1; Supplementary Fig. 1). The procedures for the structure determination of these complexes were based on non-crystallographic symmetry averaging and step-by-step phase extension^20^ (Methods). All three receptor analogues were observed to bind near the ‘eastern end' of the canyon (Fig. 2). The EV-D68 canyon is unusually shallow and narrow compared with other picornaviruses that bind Ig-like receptor molecules^8^,^20^. In all three structures the Neu5Ac moiety is well accommodated in a wide crater formed by VP1 and VP3 within the same protomer^6^ (Fig. 2; Supplementary Fig. 2).

**Figure 3:**
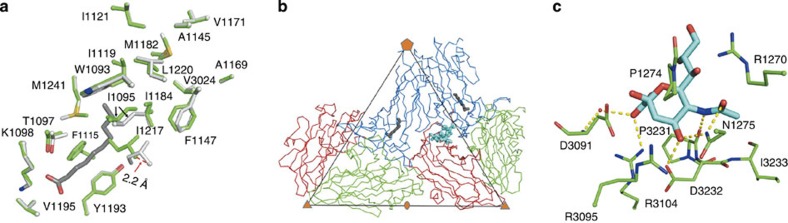
Binding of sialylated receptor analogues to EV-D68 displaces the pocket factor. (**a**) Superimposition of the amino acids lining the VP1 hydrophobic pocket in the native structure (light grey) and the receptor bound complex (green). The pocket factor in the native structure is coloured grey. (**b**) Two protomers of the EV-D68 capsid are represented as Cα backbones with VP1, VP2 and VP3 coloured blue, green and red, respectively. The pocket factor (native structure) and the receptor analogue (receptor bound complex) are coloured grey and cyan, respectively. (**c**) The sialic acid moiety (Neu5Ac) (cyan carbon atoms) interacts with surrounding amino acids (green carbon atoms). A water molecule is represented by a red sphere. Dash lines indicate polar interactions. Oxygen, nitrogen and sulfur atoms in (**a**,**c**) are colored red, dark blue and yellow, respectively.

The sialic acid moiety (Neu5Ac) of these ligands is stabilized by a series of interactions provided by the surrounding residues Arg3104, Asp3232, Pro3231, Asn1275, Pro1274, Arg1270, Asp3091, Arg3095 and Ile3233 (Fig. 3) (The EV-D68 amino acid numbering system is based on the Fermon strain amino acid sequence. Residues in VP1, VP2 and VP3 are defined by adding 1,000, 2,000 and 3,000 to their sequence number). Thus, like other viral attachment proteins that bind terminal Neu5Ac (ref. 31), EV-D68 makes polar interactions with the carboxylate group and the acetamido group nitrogen atom of Neu5Ac (Fig. 3). The residues that interact with Neu5Ac in EV-D68 are conserved except for an Arg to Lys change at position 1270 among 51 EV-D68 isolates collected from sources on four continents between 1962 and 2014 (Supplementary Table 2). These isolates have been classified as belonging to three lineages, although lineage 1 was the most dominant. It is, therefore, significant that these residues are also conserved in EV-D70, which is known to bind sialic acid as a cellular receptor^27^. However, enteroviruses that bind Ig-like molecules in the canyon have quite different kinds of residues at the sialic acid binding site of EV-D68 (Supplementary Table 3). In addition, Coxsackievirus A24, which uses sialic acid as a receptor, also has different kinds of residues at the EV-D68 sialic acid binding site, suggesting that sialic acid would bind to a different site on Coxsackievirus A24 than on EV-D68. This is consistent with the fact that Coxsackievirus A24 binds to sialic acid at a site near each five-fold axis, as shown by a previous study on the structures of Coxsackievirus A24 complexed with sialic acid or sialylated glycans^32^. Moreover, the infectivity of the EV-D68 strain (US/MO/14-18947) from the 2014 US outbreak (which has a lysine in position 1,270) was significantly reduced in neuraminidase-treated RD cells as compared with non-treated RD cells (Supplementary Fig. 3), suggesting that sialic acid might be a common receptor for a broad spectrum of EV-D68 strains.

### Receptor specificity

Different conformations were observed between the 6′SLN and 3′SLN receptor analogues that might explain why EV-D68 preferentially recognizes sialic acid receptors with an α-2,6 linkage^28^ (Supplementary Fig. 4). These are consistent with the fact that 6′SLN prevents EV-D68 attachment and inhibits killing of RD cells more efficiently than does 3′SLN (Fig. 1g,h). This suggests that EV-D68 might have a tropism towards the human upper respiratory tract, where α-2,6 linked sialic acid molecules are more abundant and the temperature is optimal for EV-D68 growth than in the lower respiratory tract^2^.

The receptor analogues used here are trisacchrides and presumably could be a component of the glycoconjugate on the authentic receptor that might be a glycoprotein or glycolipid. However, a previous glycan array analysis^28^ used 3′SLN and 6′SLN covalently linked to bovine serum albumin (BSA) through the GlcNAc moiety. This analysis found that the Fermon strain has a limited binding affinity for 3′SLN linked to BSA compared with 6′SLN linked to BSA. In the present study, structures of EV-D68 complexed with these sialylated glycans showed that 3′SLN adopts a linear conformation such that it is lying against the eastern rim of the virus canyon (Supplementary Fig. 4; Supplementary Movie 1). Thus, if 3′SLN linked to BSA were to bind to the same binding site, the virus would impose a large steric hindrance that would not accommodate the BSA. In contrast, 6′SLN (as also 6′SL) adopts a bent conformation such that the GlcNAc moiety, which is furthest away from the Neu5Ac moiety, is well clear of the virus surface which, therefore, permits the presence of BSA (Supplementary Fig. 4; Supplementary Movie 1). Therefore, steric hindrance might limit the binding of the virus to α-2,3 but not α-2,6 linked sialic acid on the authentic sialylated receptors.

### Sialic acid binding causes ejection of the pocket factor

**Figure 4:**
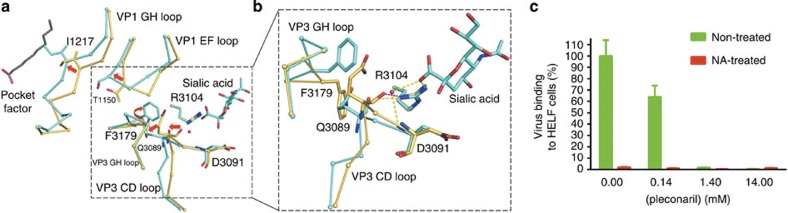
Competition between the sialic acid receptor and the pocket factor. (**a**) The conformational changes of the virus when sialylated receptor analogues bind the virus and eject the pocket factor. Amino acids in the native and in the receptor bound structures are shown in yellow and cyan, respectively. A water molecule is shown as a red sphere. Dash lines represent polar interactions. Red arrows indicate movements of the four loops. (**b**) Enlarged component of marked region in (**a**) shown in a slightly different orientation. (**c**) Preincubation of EV-D68 with pleconaril inhibits viral attachment onto non-treated HELF cells. Data are represented as mean±s.d. Experiments were done in triplicate.

The r.m.s.d. between all the equivalent atoms of the EV-D68 native structure and the EV-D68 bound with any of the three receptor analogues is about 0.4 Å (Supplementary Table 4). Nearly all main chain atoms that were displaced by more than four r.m.s.d. were in the loops that form the connecting region between the sialic acid binding site and the VP1 hydrophobic pocket. The same conformational changes occurred in each of the three complexes. The conformational changes included the VP1 GH loop located at the boundary between the VP1 hydrophobic pocket and the canyon. Similar changes in the VP1 GH loop occur when CD155 binds to poliovirus^14^. In particular, the Cα atom of residue Ile1217 moves 2.2 Å into the pocket (Fig. 3). This results in a conformation of the VP1 GH loop much like that in the rhinovirus 14 structure where no pocket factor is present^6^. Thus binding of sialylated receptor analogues onto EV-D68 causes a partial collapse of the pocket, leading to the displacement of the pocket factor (Fig. 4). However, in these sialylated receptor analogue bound structures, the VP4 density is still present and the particle size is not altered, indicating that sialylated glycan binding of EV-D68 at room temperature represents an initial event of the viral entry process. In contrast to the conformational changes that occur when a sialic acid receptor binds into the canyon, binding of glycan receptors to other binding sites (Supplementary Table 5) on picornaviruses do not cause further conformational changes.

The contact region for all three receptor analogues is the VP1 C terminus, VP3 C terminus and VP3 CD loop. This site is ∼28 Å from the VP1 hydrophobic pocket in the same protomer and ∼30 Å away from the VP1 hydrophobic pocket in a neighbouring protomer (Fig. 3). In contrast, all Ig-like molecules interact directly with the VP1 GH loop in the canyon (Supplementary Fig. 5). Thus, binding of the sialylated receptor analogues displaces the pocket factor through long range structural rearrangements of the virus. The region connecting the sialic acid receptor binding site and the VP1 hydrophobic pocket is formed by the VP3 CD loop, the VP3 GH loop, the VP1 EF loop and the VP1 GH loop. These loops undergo the only significant conformational changes mentioned above (Supplementary Table 4). Thus the signal of receptor binding must be transmitted to the hydrophobic pocket through the structural rearrangements of these loops (Fig. 4a,b). These conformational changes might be initiated by charge repulsion between the negatively charged carboxylate group of Neu5Ac and the main chain carbonyl group of Gln3089. In partial agreement with this mechanism, two of the five mutations that resulted in the resistance of EV-D70 to neuraminidase treatment of HeLa cells occur in the VP1 EF loop and GH loop^33^. Furthermore, the pocket factor was missing and the hydrophobic pocket was collapsed in A particles of other enteroviruses^34^, while three of the four loops mentioned above undergo dramatic conformational changes during virus expansion to A particle in the uncoating process^35^. In summary, binding of sialic acid receptor analogues to EV-D68 ejects the pocket factor and therefore presumably destabilizes the virus to facilitate viral uncoating.

Furthermore, in analogy with the interaction of EVs with Ig-like receptors^10^, the structure of EV-D68 complexed with sialylated trisaccharides suggests that the receptor molecule and the pocket factor compete to bind the virion in the initial step of EV-D68 entry into host cells. Consistent with this is that pleconaril inhibits viral attachment to HELF cells (Fig. 4c) because the bound pleconaril blocks the conformational change of the virus that favours sialic acid binding.

## Discussion

In the present study, a recent clinical isolate of EV-68 (US/MO/14-18947 from the 2014 US outbreak) was shown to have sialic acid dependency, as is the case for the prototype Fermon strain and another early strain isolated in the 1970s (ref. 36). Furthermore, RD and HeLa cells were found to be susceptible to infection by both the Fermon and US/MO/14-18947 strains. In particular, these two strains exhibited similar growth kinetics when using RD cells (Fig. 1; Supplementary Fig. 3). Thus the similarities between these two strains suggest that the results described here might be equally applicable to at least some of the recent isolates. However, the Fermon strain is likely to have undergone some changes since its isolation in 1962 due to a lengthy passage history, which may have changed its receptor binding properties and/or fitness in susceptible cell lines^37^. Therefore, it is possible that some recent strains may have potentially altered receptor usage. It is also probable that some recent strains may not replicate as efficiently as the Fermon strain in cells lines such as RD, HeLa and HELF cells examined here.

Although EV-D68 causes mainly respiratory infections, some recent isolates were associated with neurological illness such as acute flaccid myelitis^38^,^39^,^40^. These recent clinical isolates are all in the same clade as US/MO/14-18947 isolated from a patient with respiratory illness but who did not have a neurological disease^39^. The amino acid sequences of the structural proteins of these isolates share ∼99% sequence identity to that of US/MO/14-18947. Furthermore, the 51 strains used for sequence alignment, as mentioned above, include strains that were associated with neurological diseases. This suggests that these strains might also be sialic acid dependent, consistent with the fact that most EV-D68 associated acute flaccid myelitis cases had respiratory infections^39^.

In summary, the structural and functional analyses showed that sialic acid is a functional cellular receptor for EV-D68. The prevalence of sialic acid in the human respiratory tract might allow efficient replication of EV-D68 that leads to human respiratory infection. Despite chemical and structural differences from the Ig-like receptors, the sialic acid receptor binds to the virus canyon and causes the expulsion of the pocket factor, suggesting that the enterovirus canyon is a sensor for receptors. Thus, the canyon transmits signals initiated by receptor binding to release the pocket factor, regulating the conformational state of EVs from being stable for virus transmission to being unstable during virus entry.

## Methods

### Viruses and cells

HeLa cells (H1-HeLa cells, ATCC CRL-1958) and human rhabdomyosarcoma (RD, ATCC CCL-136) cells were purchased from ATCC. Human embryonic lung fibroblast (HELF) cells and the prototype strain of EV-D68 were kindly supplied by Dr M. Steven Oberste at the Centers for Disease Control and Prevention (USA). CMAS knockout HAP1 cells were obtained from Haplogen GmbH (Vienna, Austria). RD and HELF cells were maintained in Dulbecco's Modified Eagle Medium (Sigma-Aldrich) supplemented with 10% fetal bovine serum (FBS, Sigma-Aldrich) and nonessential amino acid (NEAA, Life Technologies), while HeLa cells were maintained in minimum essential medium (Life Technologies), 10% FBS and NEAA. CMAS knockout HAP1 cells, a near-haploid human cell line that was derived from the human myeloid leukaemia cell line KBM7, were grown in Iscove's Modified Dulbecco's Medium (Life Technologies) supplemented with 10% FBS. An isolate of EV-D68 from the 2014 outbreak in the United States, US/MO/14-18947 (GenBank: AIS73051.1), was provided by Dr M. Steven Oberste through BEI Resources, National Institute of Allergy and Infectious Diseases, National Institute of Health. All EV-D68 stock was propagated in RD cells and stored at −80 °C.

### Virus production and purification

The EV-D68 prototype strain^41^ (Fermon CA62-1, GenBank: AY426531.1) was used for structural studies in this work. The virus was grown in RD cells at 33 °C using an multiplicity of infection (MOI) of ∼0.01. A mixture of supernatant and cells was collected at around 3 days post infection and was then spun down. The resultant cell debris was subject to multiple cycles of freezing and thawing, which was followed by homogenization and centrifugation. All supernatant was pooled up and pelleted using a Ti50.2 rotor. The pellets were resuspended in 250 mM HEPES, 250 mM NaCl (pH 7.5) and treated with 0.01 mg ml^−1^ DNAse (Sigma-Aldrich), 7.5  mg ml^−1^ RNase (Sigma-Aldrich) and 0.8  mg ml^−1^ trypsin (Sigma-Aldrich) in a sequential manner. On another round of centrifugation, the resultant pellets were resuspended and applied to a potassium tartrate gradient (10–40%, w/v) in 250 mM HEPES, 250 mM NaCl (pH 7.5) using a SW 41 rotor. A bluish band in the middle of the tube, which corresponds to full particles, was extracted. Multiple rounds of buffer exchange was then conducted to remove potassium tartarate in the sample.

### Crystallization and soaking and data collection

Cubic-looking crystals (∼0.1 mm in each dimension) were grown within 3 days at room temperature using the hanging vapour diffusion technique. A drop of 0.5 μl of virus (2–3  mg ml^−1^ in phosphate buffer saline) and 0.5 μl of reservoir solution (3.5 M sodium formate, 0.1 M sodium acetate, pH 4.5) was used for EV-D68 crystallization. Crystal soaking experiments were performed in a soaking solution that contains a given concentration of a sialylated trisaccharide in reservoir solution at room temperature for ∼8 h. The concentration used for 3′-sialyl-*N*-acetyl-lactosamine (3′SLN, V-Labs Inc.), 6′-sialyllactose (6′SL, V-Labs Inc.) and 6′-sialyl-*N*-acetyl-lactosamine (6′SLN, V-Labs Inc.) were 10, 20 and 10 mM, respectively. Crystals were cryoprotected in the aforementioned soaking solution containing glycerol (with gradually increased concentration from 0 to 20% (v/v)) and then flash-frozen in liquid nitrogen. X-ray diffraction data on single crystals were collected at 100 K at beamline 14-BM-C of the advanced photon source. The detector was an ADSC Q315 charge coupled device. The oscillation angle was 0.2°. Data collection statistics are summarized in Supplementary Table 1.

### Structure determinations

Procedures for determining all three complex structures were the same, as described below. X-ray diffraction data were processed using the program HKL2000 (ref. 42). The space group for all crystals was I222. Calculation of the Matthew's coefficient indicated that there are two particles present in each unit cell. Thus the particles must be sitting on 222 symmetry positions. Calculation of a self-rotation function differentiated between the two possible 90° related orientations of the particle. The atomic model of EV-D68 (PDB accession number 4WM8) excluding the pocket factor and water molecules was employed to calculate phases to 8.0 Å resolution. Phases were improved by 10 cycles of 15-fold non-crystallographic symmetry (NCS) averaging using the program AVE (ref. 43). The mask defining the volume of NCS averaging was generated by selecting all grid points within a radius of 5 Å from each atom of the initial model using the program MAMA^44^. Phases were then iteratively extended to the resolution limit of the data set using a step size of (1/a)Å^−1^, where a is the length of the cell dimension in the a direction. Each step was followed by five cycles of NCS averaging at the current resolution limit. Manual model building was performed using the program Coot^45^. The resultant structure was subjected to atomic position and B factor refinement with simulated annealing by applying NCS constraints using the program CNS^46^. Water molecules were finally added and checked with the program Coot. All atomic models were validated based on the criteria of Molprobity^47^ using the program package Phenix^48^. Other calculations were done using the CCP4 program suite^49^. The structures of symmetry related icosahedral asymmetric units were generated using VIPERdb (ref. 50). Figures were made using Pymol (http://www.pymol.org/). Roadmaps were generated using the program RIVEM^51^.

### Plaque assays

A series of 10-fold dilution were made for each sample. The resultant sample with a given dilution was added to confluent HeLa cells in a six-well plate. After virus absorption at room temperature for 1 h, cells were covered with an overlay of 0.9% agarose in MEM and 5% FBS per well. Plates were allowed to be incubated at 33 °C for ∼5 days. Plaques were visible and counted after neutral red or crystal violet staining.

### Virus infection assays

Each of the three cell lines, HeLa, RD and HELF cells, grown to ∼90% confluency in 24-well plates were treated with either 100 mU ml^−1^ neuraminidase from *Clostridium perfringens* (Sigma-Aldrich) in Dulbecco's Modified Eagle's Medium (DMEM) and 1XNEAA (solution A) or solution A only at 37 °C for 1 h. Cells were then washed with DMEM once and infected with the EV-D68 strain Fermon CA62-1. The MOI for HeLa, RD and HELF cells were 0.3, 0.001 and 0.01, respectively. After absorption at 33 °C for 1 h, the inoculum was removed and cells were washed twice with DMEM. Cells were incubated at 33 °C in a medium of DMEM supplemented with 5% FBS and NEAA for 0, 24, 48 or 72 h post attachment. Samples were collected at each of these time points. These samples were freeze-thawed multiple times and then subjected to virus titer determination using plaque assays. For the EV-D68 strain US/MO/14-18947, infection experiments on RD cells were performed in the same way as described above for the Fermon prototype strain. All experiments were done in at least triplicate.

For virus infection of HAP1 cells, HAP1 WT and CMAS-KO cells were seeded in a 96-wells plate at 20.000 cells per well. Cells were treated with neuraminidase (from *C. perfringens,* New England Biolabs) 1:50 diluted in Iscove's Modified Dulbecco's Medium for 30 min at 37 °C. After neuraminidase was removed, EV-D68 (strain Fermon CA62-1) was added to the cells at an MOI of 1 and incubated for 1 hr at 37 °C. Virus was removed and neuraminidase (or only medium) was added again to the cells. Cells were frozen immediately (*T*=0) or 10 h after addition of the virus. Virus titres (expressed as TCID_50_ ml^−1^) were determined by an end-point dilution assay using RD cells at 33 °C.

### Competition experiments

Cytopathic effect inhibition assays^52^ were conducted in the following way. For each of the three glycans, 6′SL, 6′SLN and 3′SLN, EV-D68 (strain Fermon CA62-1) diluted in DMEM was incubated with a series of concentrations of glycans ranging from 0 to 4 mM at 33 °C for 1 h, where 0 mM was designated as the non-treated virus control. For each given concentration of glycan, a control was performed in which DMEM was incubated with glycan. The resultant mixture was added into ∼90% confluent RD cells in each well of a 96-well plate. The MOI was 0.001. After virus absorption at 33 °C for 1 h, the inoculum was removed and cells were washed twice with DMEM. Cells were allowed to be incubated in DMEM supplemented with 5% FBS and NEAA at 33 °C for 3 days, which ensured that almost all cells became detached in the non-treated virus control. After removal of the medium, cell viability was assayed using a Quick Cell Proliferation Colorimetric Assay Kit (BioVision). The optical density for each well was recorded at a wavelength of 450 nm using a SpectraMax M5 Microplate Reader (Molecular Devices). Per cent inhibition was calculated as (ODcg-ODvg)/(ODc-ODv) * 100%, where ODcg and ODvg are the optical density of the uninfected and infected cell cultures treated with a given concentration of glycan, respectively. ODc and ODv are the optical density of the uninfected and infected cells treated with water only but no glycan, respectively. All experiments were done in triplicate.

### Viral attachment assays

RD cells with ∼90% confluent in 24-well plates. These were treated with 100 mU ml^−1^ neuraminidase in solution A or solution A only at 37 °C for 1 h. After washing with cold DMEM once, each well was treated with 500 μl of DMEM containing 1% bovine serum album (blocking solution) at 4 °C for ∼15 min. The blocking solution was then removed and purified EV-D68 (strain Fermon CA62-1) diluted in cold DMEM was added onto cells with an MOI of 10. After incubation at 4 °C for 1 h, the wells were washed three times with cold DMEM to remove unbound virus.

Competition assays were performed using purified virus incubated with either 3′SLN or 6′SLN in a series of concentrations ranging from 0 to 3.2 mM, where 0 mM represents the virus only control in which virus was incubated with water alone. The resultant mixture was cooled down on ice and added to ∼90% confluent RD cells in a 24-well plate after each well was blocked with the blocking solution as described above. The MOI was 10. After incubation at 4 °C for 1 h, the wells were washed three times with cold DMEM to remove unbound virus.

To test the effect of pleconaril on EV-D68 attachment, purified virus was incubated with pleconaril in a series of concentrations ranging from 0 to 14 μM at 33 °C for 1 h, where 0 μM represents the virus only control in which the virus was incubated with only dimethyl sulfoxide . The resultant mixture was cooled down on ice and added to ∼90% confluent HELF cells in a 24-well plate right after blocking each well with the blocking solution as mentioned above. The MOI was 20. After incubation at 4 °C for 1 h, the wells were washed three times with cold DMEM to remove unbound virus. Similar results were obtained when the virus was incubated with pleconaril at 4 °C overnight.

For all virus attachment experiments, the RNA in each well was extracted using an RNeasy Mini Kit (Qiagen) per manufacturer's protocol. The viral RNA of the bound virus in each sample was quantified using a quantitative real-time reverse transcription PCR (RT-PCR) protocol. All experiments were done in triplicate.

### Quantitative real-time RT-PCR

Quantitative real-time RT-PCR was performed using a Superscript III Platinum SYBR Green One-step qRT-PCR Kit with ROX (Life Technologies) on an ABI 7300 real-time PCR system. A 25-μl reaction mixture containing RNA template, SYBR green reaction mix, SuperScript III RT/Platinum Taq Mix, primers and RNAse-free water was used, where the forward and reverse primers targeting the EV-D68 5′-untranslated region were EQ-1 (5′-ACATGGTGTGAAGAGTCTATTGAGCT-3′) and EQ-2 (5′-CCAAAGTAGTCGGTTCCGC-3′) (ref. 53). The thermal profile was 50 °C for 3 min, 95 °C for 5 min followed by 40 cycles at 95 °C for 15 s and at 60 °C for 30 s. GAPDH, a housekeeping gene, was used as an internal control for normalization purpose. The primers for GAPDH were 5′-CCCACTCCTCCACCTTTGACG-3′ (forward) and 5′-CACCACCCTGTTGCTGTAGCCA-3′ (reverse). The relative levels of EV-D68 RNA in different samples were determined using a comparative 2^−ΔΔCT^ method^54^.

## Additional information

**Accession codes:** Coordinates for EV-D68-6′SL, EV-D68-3′SLN and EV-D68-6′SLN have been deposited with the Protein Data Bank under accession numbers 5BNN, 5BNP and 5BNO, respectively.

**How to cite this article:** Liu, Y. *et al.* Sialic acid-dependent cell entry of human enterovirus D68. *Nat. Commun.* 6:8865 doi: 10.1038/ncomms9865 (2015).

## Supplementary Material

Supplementary Information Supplementary Figures 1-5, Supplementary Tables 1-5 and Supplementary References

Supplementary Movie 1 Steric hindrance imposed by the virus on α2,3 but not α2,6 sialylated receptors.
