# Supplementary material for: Sialic acid-dependent cell entry of human enterovirus D68
Source: Nat Commun. 2015 Nov 13;6:8865. doi: 10.1038/ncomms9865 (PMC4660200; doi:10.1038/ncomms9865)
Supplement: Supplementary Information — Supplementary Figures 1-5, Supplementary Tables 1-5 and Supplementary References [file ncomms9865-s1.pdf]

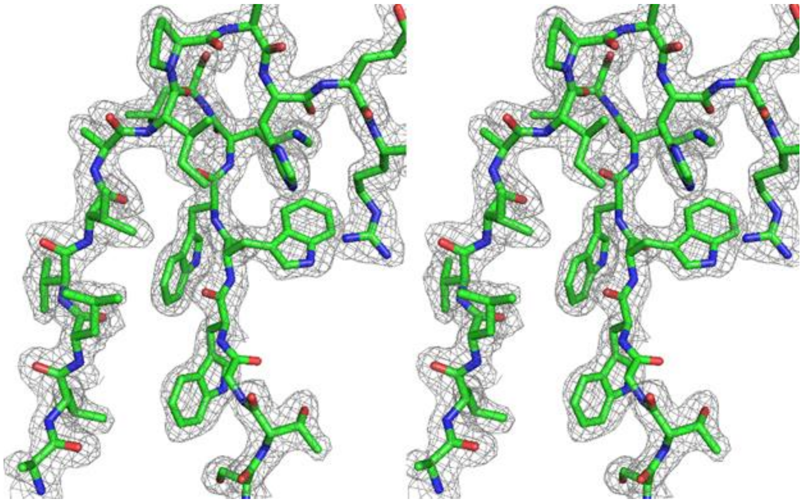

Supplementary Figure 1. Representative non-crystallographic symmetry (NCS) averaged $2F_o$ – F_c electron density map for an EV-D68-sialylated receptor analogue complex at a contour level of 1.5 sigma. Fc is the structure factor determined by back Fourier transforming the previously NCS averaged map. The figure shows a stereo view of the residues 2075-STGWWWKL-2082 and residues 2121-ALLVVAIPEHQR-2132.

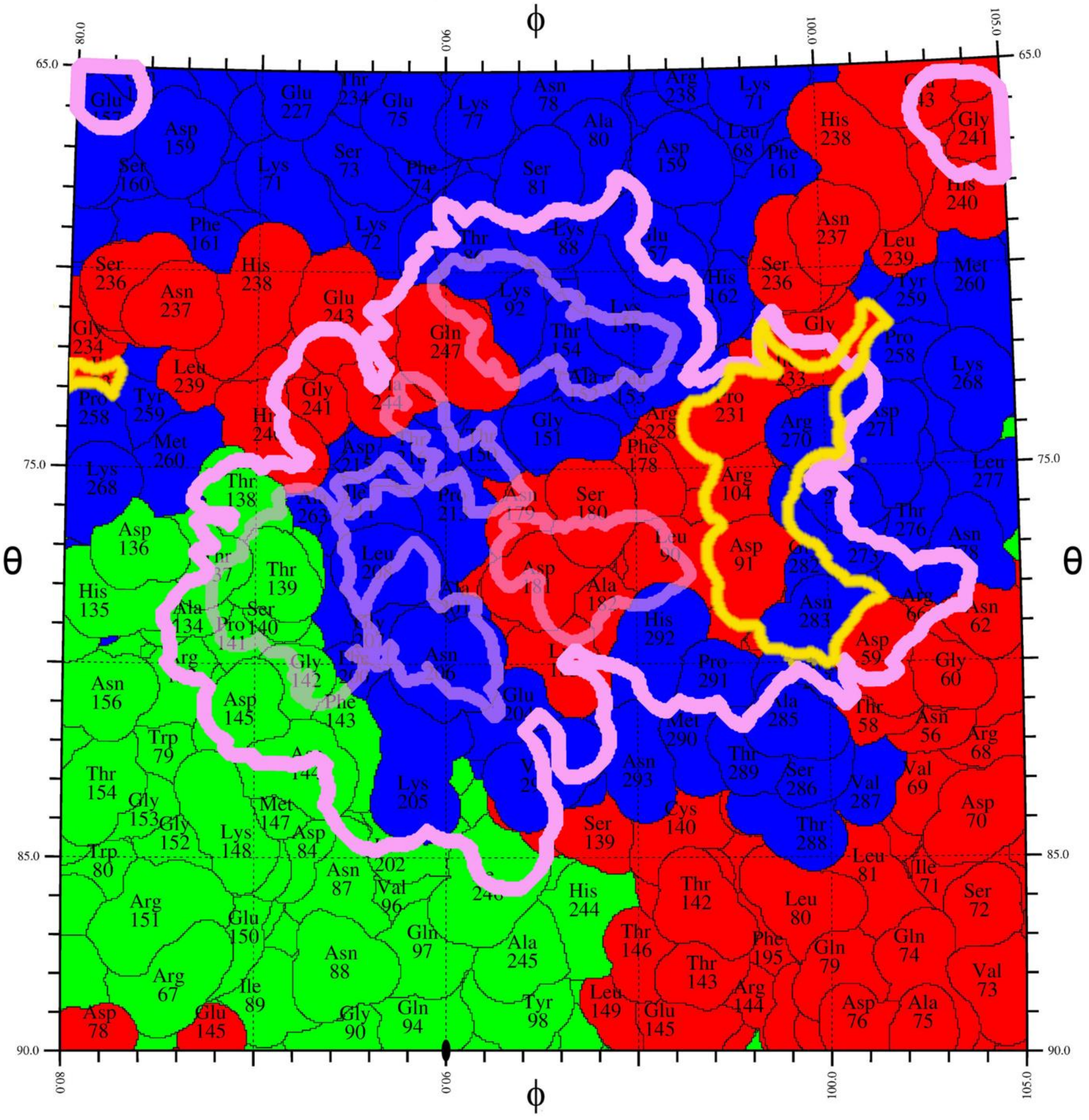

Supplementary Figure 2. A close-up view of the footprints of Ig-like receptors and sialylated receptor analogues. The footprint of Ig-like receptors on five EVs: intercellular adhesion molecule-1 for human rhinovirus 14[1], human rhinovirus 16[1] and coxsackievirus A21[2], CD155 for poliovirus 1[3], coxsackievirus and adenovirus receptor for coxsackievirus B3[4] are

superimposed and placed onto the EV-D68 surface as in **Fig. 2b**. Shown also is the identity of the EV-D68 surface amino acids.

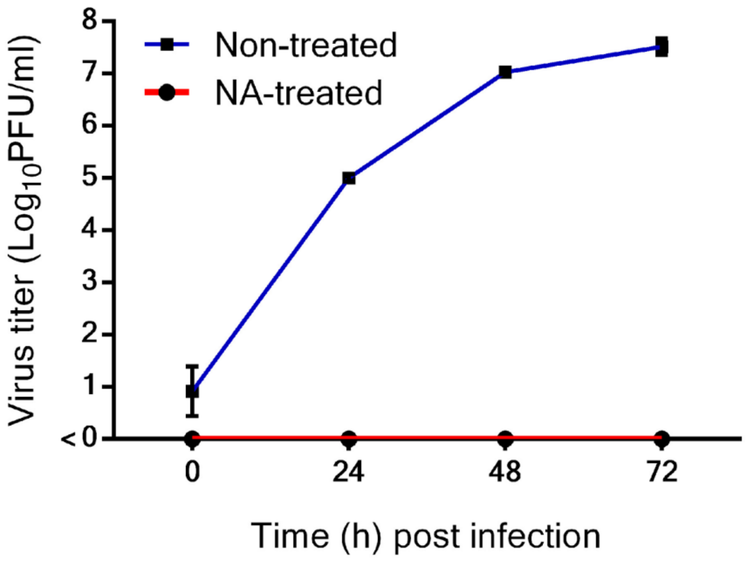

Supplementary Figure 3. Neuraminidase treatment of RD cells inhibits the infectivity of a current EV-D68 strain (US/MO/14-18947). Data are represented as mean ± SD. Experiments were done in at least triplicate.

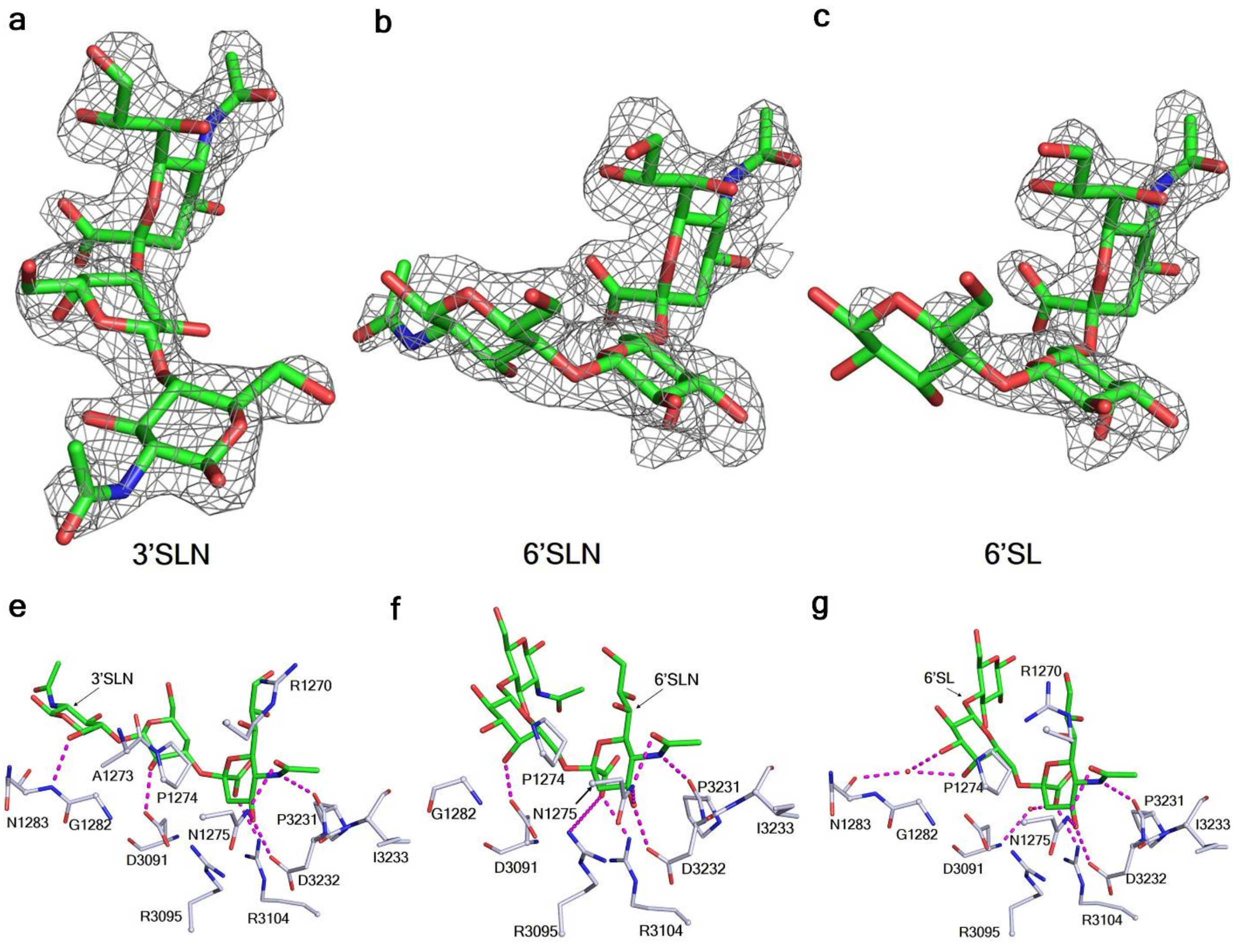

Supplementary Figure 4. Receptor specificity. The sialylated trisaccharides 6'SL (**a**, **d**), 6'SLN (**b**, **e**), and 3'SLN (**c**, **f**) are colored using green for carbon atoms, red for oxygen atoms and blue for nitrogen atoms. The top row (**a**, **b** and **c**) shows these compounds in their difference map densities all oriented to place the icosahedral two-fold axis perpendicular to the page. Shown are the non-crystallographic symmetry averaged Fr-Fn difference map (at a contour level of 3.0 sigma, grey) densities, where Fr and Fn are the structure factors for the receptor analogue bound structure and the native virus structure, respectively. The lower three panels (**d**, **e** and **f**) show their interaction with the virus, oriented to resolve the projected atoms. Dashed lines indicate polar interactions. Water molecules are shown as red spheres.

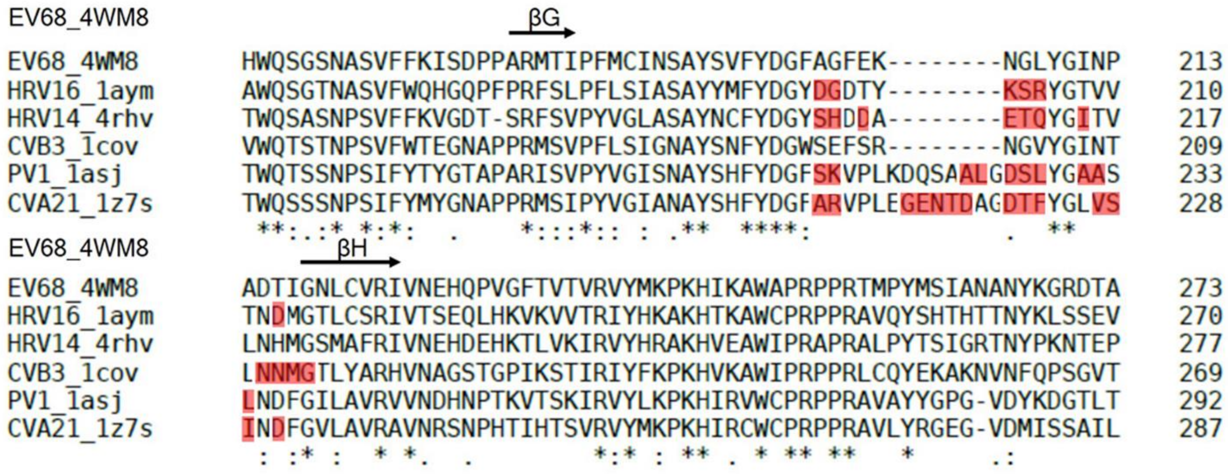

Supplementary Figure 5. The VP1 GH loop is invariably in the footprint of Ig-like receptors used by EVs. Residues in the contact region are highlighted. Black arrows represent β-strands.

Supplementary Table 1 Data collection and refinement statistics

	EV68 – 6'SL	EV68 – 6'SLN	EV68 – 3'SLN
**Data collection**			
Wavelength (Å)	0.9787	0.9787	0.9787
Oscillation angle (°)	0.2	0.2	0.2
No. of crystals (No. of frames)	1 (150)	1 (170)	1 (160)
Space group	*I*222	*I*222	*I*222
Cell dimensions			
a, b, c (Å)	325.6, 347.1, 356.4	325.7, 347.4, 356.5	325.8, 347.2, 356.9
α, β, γ (°)	90.0, 90.0, 90.0	90.0, 90.0, 90.0	90.0, 90.0, 90.0
Resolution (Å)	50.0-2.32 (2.40-2.32)*	50.0-2.15 (2.23-2.15)	50.0-2.15 (2.23-2.15)
No. of unique reflections	526043 (55652)	803405 (80335)	823936 (84149)
Completeness (%)	61.4 (65.3)	74.6 (75.0)	76.3 (78.3)
Redundancy	1.9 (1.8)	1.8 (1.6)	1.7 (1.6)
*R*merge[†]	0.175 (0.722)	0.152 (0.865)	0.124 (0.529)
$I/\sigma(I)$	4.16 (1.04)	5.37 (0.89)	6.05 (1.45)
**Refinement**			
Resolution (Å)	50.0-2.32 (2.40-2.32)	50.0-2.15 (2.23-2.15)	50.0-2.15 (2.23-2.15)
No. of reflections	525999 (55490)	802817 (79585)	823388 (84396)
R factor	0.246 (0.333)	0.254 (0.327)	0.244 (0.314)
Correlation coefficient[‡]	0.886	0.922	0.921
No. of atoms	6610	6647	6679
Protein	6296	6303	6317
Ligand	43	46	46
Water	271	298	316
RMSD bond (Å)	0.006	0.006	0.006

RMSD angle (°)	1.378	1.393	1.392
Mean *B*-factor ($Å^2$)	19.3	25.1	20.0
Ligand *B*-factor ($Å^2$)	51.6	70.6	63.7
Ramachandran plot[§]			
Favored (%)	96.1	96.2	95.9
Allowed (%)	3.9	3.7	4.1
Outliers (%)	0.0	0.1	0.0

*Values in parentheses represent the highest resolution shell

[†] $R_{\mathrm{merge}} = \sum_{hkl} \sum_i |I_{\mathrm{i}}(hkl) - \langle I(hkl) \rangle| / \sum_{hkl} \sum_i I_{\mathrm{i}}(hkl)$

[‡] Correlation coefficient of F_o and F_c after the convergence of NCS averaging, where F_c are the structure factor amplitudes determined by back Fourier transforming the NCS averaged map

[§] According to the criteria of Molprobity[5]

Supplementary Table 2. Temporal and geographical origin of 51 EV-D68 isolates[*]

GenBank Accession number	Country	Collection date (year)	Genetic group (lineage) [†]	Genetic group (clade) [‡§]
AY426531.1	USA	1962	prototype	prototype
EF107098.1	France	2006[‖]	2	C
KF726085.1	China	2008	3	A
JX101846.1	USA	2009	3	A
JX070222.1	New Zealand	2010	3	A
AB601882.2	Japan	2010	2	C
AB601883.2	Japan	2010	2	C
AB601884.1	Japan	2010	2	C
AB601885.1	Japan	2010	2	C
KM892499.1	USA	2013	1	B
KM892500.1	USA	2013	3	A
KP100793.1	USA	2013	1	B
KM851225.1	USA	2014	1	B
KM851226.1	USA	2014	1	B
KM851227.1	USA	2014	1	B
KM851228.1	USA	2014	1	B
KM851229.1	USA	2014	1	B
KM851230.1	USA	2014	1	B
KM851231.1	USA	2014	3	A
KM881710.2	USA	2014	1	B
KM892501.1	USA	2014	1	B
KP100792.1	USA	2014	1	B
KP100794.1	USA	2014	1	B
KP100795.1	USA	2014	1	B
KP100796.1	USA	2014	1	B
KP126911.1	China	2014	1	B
KP240936.1	USA	2014	1	B
KP745751.1	USA	2014	1	B

KP745752.1	USA	2014	1	B
KP745753.1	USA	2014	1	B
KP745754.1	USA	2014	1	B
KP745755.1	USA	2014	1	B
KP745756.1	USA	2014	1	B
KP745757.1	USA	2014	1	B
KP745758.1	USA	2014	1	B
KP745759.1	USA	2014	1	B
KP745760.1	USA	2014	1	B
KP745761.1	USA	2014	1	B
KP745762.1	USA	2014	1	B
KP745763.1	USA	2014	1	B
KP745764.1	USA	2014	1	B
KP745765.1	USA	2014	1	B
KP745766.1	USA	2014	1	B
KP745767.1	USA	2014	1	B
KP745768.1	USA	2014	1	B
KP745769.1	USA	2014	1	B
KP745770.1	USA	2014	1	B
KP114662.1	Canada	2014	1	B
KP114663.1	Canada	2014	1	B
KP114664.1	Canada	2014	1	B
KP114665.1	Canada	2014	1	B

[*] Strains that have the complete sequence of the capsid coding region as of May 2015

[†] Imamura et al.[6]

[‡] Tokarz et al.[7]

[§] The relationship between the two classification systems mentioned above was reported by Meijer et al.[8]

[ǀ] Submission date of the sequence

Supplementary Table 3 Alignment of amino acids that interact with sialic acid in EV-D68 with the equivalent amino acids in other EVs*

Receptor	Virus	Species	Amino acids[†]								
			1270	1274	1275[‡]	3091	3095	3104[‡]	3231[‡]	3232[‡]	3233
Sialic acid	EV-D68_4WM8[§]	EV-D	R	P	N	D	R	R	P	D	I
Sialic acid	EV-D70[††]	EV-D	R	P	N	E	R	R	P	D	I
Scavenger receptor B2[¶]	EV-A71_3ZFE	EV-A	N	P	T	D	Q	G	S	H	I
CAR[ǀ]	Coxsackievirus B3_1COV[#]	EV-B	S	T	T	S	S	N	P	F	I
CD155	Poliovirus 1_1ASJ[#]	EV-C	-[☆]	-[☆]	-[☆]	D	S	N	T	H	I
ICAM-1[**]	Coxsackievirus A21_1Z7S[#]	EV-C	-[☆]	-[☆]	-[☆]	D	S	N	P	H	I
Sialic acid	Coxsackievirus_A24_4Q4W	EV-C	-[☆]	-[☆]	-[☆]	D	Q	N	N	H	I
ICAM-1[**]	Human rhinovirus 16_1AYM[#]	RV-A	S	H	N	-[☆]	A	S	D	L	H
ICAM-1[**]	Human rhinovirus 14_4RHV[#]	RV-B	N	V	I	-[☆]	K	Q	Q	T	I

[*]Viruses that belong to the species EV-D and bind to sialic acid are highlighted in green
[†]Numbering based on the amino acid sequence of the EV-D68 Fermon CA 62-1 strain
[‡]The amino acid provides polar interaction with the Neu5Ac moiety through side chain or main chain
[§]Virus name_PDB accession number
[ǀ]CAR: Coxsackievirus and adenovirus receptor
[¶]Scavenger receptor B2 was proposed to bind to the EV-A71 canyon[9]
[#]The virus uses an Ig-like molecule as a cellular receptor
[☆]"–" represents deletion at a given position
[**]ICAM-1: Intercellular Adhension Molecule-1
[††]Strain J670/71

Supplementary Table 4 Root mean square deviations* (r.m.s.d) between two given structures with or without a bound receptor analogue

Structure A	Structure B	Overall	3086-3091[†] (VP3 CD loop)	3178-3179 (VP3 GH loop)	1149-1153 (VP1 EF loop)	1211-1219 (VP1 GH loop)
EV-D68-6'SL	EV-D68	0.40	1.93	2.28	2.72	1.91
EV-D68-3'SLN	EV-D68	0.36	1.78	2.31	2.65	1.81
EV-D68-6'SLN	EV-D68	0.35	1.80	2.19	2.24	1.83
EV-D68-6'SL	EV-D68-3'SLN	0.10	0.24	0.05	0.21	0.20
EV-D68-6'SL	EV-D68-6'SLN	0.11	0.21	0.48	0.48	0.21
EV-D68-3'SLN	EV-D68-6'SLN	0.06	0.09	0.33	0.34	0.09

*VP1, VP2 and VP3 were used for superimposition. r.m.s.d. values (Å) were calculated using main chain atoms (N, Cα, C, O).

[†]Residues are numbered according to the amino acid sequence of the EV-D68 strain Fermon CA 62-1.

Supplementary Table 5 List of glycan receptor binding sites on picornaviruses

Virus	Bound glycan receptor	PDB accession number	Binding site	Reference
Coxsackievirus A24	Sialic acid (Neu5Ac)	4Q4X, 4Q4Y	Near five-fold axes	[10]
Foot-and-mouth disease virus	Heparan sulfate	1QQP	Near the center of protomers	[11]
Equine rhinitis A virus	3'-sialyllactose	2XBO	A shallow groove near the VP1 EF loop	[12]
Theiler's murine encephalitis virus	3'-sialyllactose	Not available	A depression formed by puff B of VP2	[13]
Enterovirus D68	3'SLN, 6'SLN, 6'SL	5BNP, 5BNO, 5BNN	Canyon	This work

Supplementary References

1 Xiao, C. *et al.* Discrimination among rhinovirus serotypes for a variant ICAM-1 receptor molecule. *J. Virol.* **78**, 10034-10044 (2004).

2 Xiao, C. *et al.* The crystal structure of coxsackievirus A21 and its interaction with ICAM-1. *Structure* **13**, 1019-1033 (2005).

3 Strauss, M. *et al.* Nectin-like interactions between poliovirus and its receptor trigger conformational changes associated with cell entry. *J. Virol.* **89**, 4143-4157 (2015).

4 Organtini, L. J., Makhov, A. M., Conway, J. F., Hafenstein, S. & Carson, S. D. Kinetic and structural analysis of coxsackievirus B3 receptor interactions and formation of the A-particle. *J. Virol.* **88**, 5755-5765 (2014).

5 Chen, V. B. *et al.* MolProbity: all-atom structure validation for macromolecular crystallography. *Acta Crystallogr. D Biol. Crystallogr.* **66**, 12-21 (2010).

6. Imamura T, Oshitani H. Global reemergence of enterovirus D68 as an important pathogen for acute respiratory infections. Rev Med Virol 25, 102-114 (2015).

7. Tokarz R, et al. Worldwide emergence of multiple clades of enterovirus 68. J. Gen. Virol. 93, 1952-1958 (2012).

8. Meijer A, Benschop KS, Donker GA, van der Avoort HG. Continued seasonal circulation of enterovirus D68 in the Netherlands, 2011-2014. Euro Surveill 19, (2014).

9 Dang, M. *et al.* Molecular mechanism of SCARB2-mediated attachment and uncoating of EV71. *Protein Cell* **5**, 692-703, doi:10.1007/s13238-014-0087-3 (2014).

10 Zocher, G. *et al.* A sialic acid binding site in a human picornavirus. *PLoS Pathog.* **10**, e1004401, doi:10.1371/journal.ppat.1004401 (2014).

11 Fry, E. E. *et al.* The structure and function of a foot-and-mouth disease virus-oligosaccharide receptor complex. *EMBO J.* **18**, 543-554 (1999).

12 Fry, E. E. *et al.* Crystal structure of equine rhinitis A virus in complex with its sialic acid receptor. *J. Gen. Virol.* **91**, 1971-1977 (2010).

13 Zhou, L., Luo, Y., Wu, Y., Tsao, J. & Luo, M. Sialylation of the host receptor may modulate entry of demyelinating persistent Theiler's virus. *J. Virol.* **74**, 1477-1485 (2000).
